# Cloning, expression, and purification of insect (*Sitophilus oryzae*) alpha-amylase, able to digest granular starch, in *Yarrowia lipolytica* host

**DOI:** 10.1007/s00253-014-6314-2

**Published:** 2014-12-31

**Authors:** Ewelina Celińska, Wojciech Białas, Monika Borkowska, Włodzimierz Grajek

**Affiliations:** Department of Biotechnology and Food Microbiology, Poznań University of Life Sciences, ul. Wojska Polskiego 48, 60-627 Poznań, Poland

**Keywords:** Amylase, Granular starch, Rice weevil, *Yarrowia lipolytica*, Recombinant protein expression

## Abstract

Raw-starch-digesting enzymes (RSDE) are of major importance for industrial applications, as their usage greatly simplifies the starch processing pipeline. To date, only microbial RSDE have gained considerable attention, since only microbial production of enzymes meets industrial demands. In this study, α-amylase from rice weevil (*Sitophilus oryzae*), the major rice pest, was cloned and expressed in *Yarrowia lipolytica* Po1g strain. The enzyme was secreted into the culture medium, and the peak activity (81 AU/L) was reached after only 29 h of culturing in 5-L bioreactors. Through simple purification procedure of ammonium sulfate precipitation and affinity chromatography, it was possible to purify the enzyme to apparent homogeneity (25-fold purification factor, at 5 % yield). The optimal conditions for the α-amylase activity were pH 5.0 and a temperature of 40 °C. The α-amylase studied here did not show any obligate requirement for Ca^2+^ ions. The recombinant α-amylase appeared to efficiently digest granular starch from pea, amaranth, waxy corn, and waxy rice.

## Introduction

The search for novel enzymes with potential applications in industrial processes is receiving constantly increasing interest from the scientific and industrial community. α-Amylases (EC 3.2.1.1, 1,4-α-d-glucan-glucanohydrolases) are one of the most important industrially relevant enzymes, with a number of applications, i.e., baking, brewing, and textile and paper industry (Gupta et al. 2003; Pandey et al. 2000; van der Maarel et al. 2002). One of the most desired traits of α-amylases is their ability to decompose raw, nonpretreated starch granules (Sun et al. 2010). Utilization of raw-starch-digesting enzymes (RSDE) allows elimination of the most energy-consuming and cost-inefficient step of starch slurry processing—gelatinization. Significant effort has been devoted to isolate raw-starch-digesting (RSD) amylases from various sources. RSD amylases are uniformly distributed among plant, animal, and microorganism species. However, only microbial RSD amylases have gained much attention, since production of enzymes in microorganisms generally meets industrial demands, like cost-/time-effectiveness, high productivity, ease of the process of modification, and optimization. Nevertheless, powerful genetic engineering methods surpass the limitations of the other sources of the enzymes and allow the production of any enzymatic activity in the preferred microbial host. Heterologous expression offers the possibility of production of potentially useful enzymes at high quantities, preferably secreted into the culture medium, which greatly facilitates their purification. Moreover, it has been reported that heterologous expression may change the properties of an enzyme, e.g., improve its thermal stability (Yuzbashev et al. 2012). RSDE have been most frequently cloned and expressed in either *Escherichia coli* or *Saccharomyces cerevisiae* hosts, and mostly genes from microbial origins have been investigated: *Aspergillus awamori* (Matsubara et al. 2004), various *Bacillus* species (Demirkan et al. 2005), *Saccharomycopsis fibuligera* (Hostinová et al. 2010), *Cryptococcus* (Iefuji et al. 1996), *Cytophaga* sp. (Jeang et al. 2002), *Rhizopus oryzae* (Li et al. 2011), and *Thermobifida fusca* (Yang and Liu 2007). The latter gene was also expressed in other hosts, namely *Pichia pastoris* (Yang et al. 2010a) and *Yarrowia lipolytica* (Yang et al. 2010b), showing superior performance of the latter host.

In this study, we cloned α-amylase (*Amy1*) gene from *Sitophilus oryzae* and expressed it in a nonconventional yeast species, *Y. lipolytica. S. oryzae* is primarily known as a major pest of stored rice, contributing to limitation of food availability for a large number of people. We assumed that high expansiveness of *S. oryzae* in cereal crops may be to some extent attributed to highly active digestive enzymes, especially amylases, synthesized by this insect. *Y. lipolytica* is a recognized system for heterologous protein expression (Barth and Gaillardin 1996; Madzak et al. 2004). Direct comparison of different expression platforms—*Y. lipolytica*, *S. cerevisiae*, *P. pastoris*, *Arxula adeninivorans*, *Hansenula polymorpha*, *Kluyveromyces lactis*, and *Schizosaccharomyces pombe* (Gellissen et al. 2005; Müller et al. 1998)—demonstrated that *Y. lipolytica* is characterized by several advantageous traits for heterologous protein expression over the other expression systems. Several detailed review papers regarding the applied strategies used vectors, and heterologous proteins expressed in *Y. lipolytica* are available in the literature (e.g., Madzak et al. 2004; Madzak and Beckerich 2013).

Not many studies on pure insect amylases can be found in the literature (e.g., Grossi de Sa and Chrispeels 1997; Titarenko and Chrispeels 2000). Usually studies are carried out in vivo or with a mixture of isolated isozymes. Isolation of relatively homogenous enzymatic preparation from small animals comprises laborious, multistep procedures, which does not meet the requirements for industrial production. Moreover, to isolate a sufficient amount of any enzyme, a number of individuals have to be used for a single isolation procedure, which may contribute to the variability of the material. Expression of enzymes in microbial hosts offers the possibility to eliminate the risk of the biological material variability within the population of differing, diploid individuals, sometimes at different life cycle stages.

This report comprises cloning and expression of *S. oryzae* α-amylase in *Y. lipolytica* cells, production of this enzyme in 5-L bioreactors, purification through affinity chromatography, and determination of digestibility of various starch species, including raw, granular starch, by this enzyme.

## Materials and methods

### Strains, media composition, and small-scale culture conditions

*Y. lipolytica* Po1g strain was used as an expression host for the recombinant *Amy1* gene. It was routinely maintained on YPD medium (g/L): yeast extract, 10; bactopeptone, 20; and glucose, 20 (agar, 20). Before any experiments, its *leu* ^*−*^ phenotype was verified on YNB medium (g/L): glucose, 20; yeast nitrogen base w/o amino acids, 6.7; and agar, 15. *E. coli* JM109 strain was used for the propagation of vectors. *E. coli* JM109 and all the derivatives were cultured in Luria-Bertani (LB) medium (g/L): yeast extract, 5; bactopeptone, 10; NaCl, 10; and agar, 15, supplemented with ampicillin (100 mg/L), when required. Flask cultivations were carried out in a rotary shaker, at 250 rpm, 30 or 37 °C for *Y. lipolytica* and *E. coli*, respectively. All the strains used in this study are listed in Table 1.

**Table 1:** Strains, vectors, and oligonucleotides used in this study

Name	Characteristics	Use	Supplier/reference
Strains			
*Yarrowia lipolytica* Po1g	Genotype: *MatA*, *leu2-270*, *ura3-302::URA3*, *xpr2-332*, *axp-2*; phenotype: *Leu* ^*−*^, *∆AEP*, *∆AXP*, *Suc+*, *pBR platform*	Parental strain-host for expression of recombinant *Amy1* gene	Yeastern Biotech Co., Ltd., Taiwan
*Yarrowia lipolytica* 1.18	Genotype: *MatA*, *leu2-270*, *ura3-302::URA3*, *xpr2-332*, *axp-2*; phenotype: *Leu+*, *∆AEP*, *∆AXP*, *Suc+*; 4 copies of pYLSC-AMY	Host for expression of recombinant *Amy1* gene	This study
*Escherichia coli* JM109	*F′(traD36*, *proAB+*, *lacIq*, *Δ(lacZ)M15)*, *endA1*, *recA1*, *hsdR17(rk−,mk+)*, *mcrA*, *supE44*, *λ−*, *gyrA96*, *relA1*, *Δ(lacproAB)*, *thi-1*	Host for routine cloning, vector propagation, assembly of a complete vector	Sigma-Aldrich
Vectors			
pGEM-T-Easy	https://pl.promega.com/resources/protocols/technical-manuals/0/pgem-t-and-pgem-t-easy-vector-systems-protocol/	Subcloning of the *Amy1* gene, sequencing	Promega Co., USA
pYLSC	pBR322 backbone, hybrid promoter (hp4d), XPR2 preregion, leucine gene (LEU2)—selection marker; AMY gene was cloned in *Sfi*I/*Kpn*I sites http://www.yeastern.com/Products.php?pid=273	Expression vector used for cloning and transformation of *Y. lipolytica* Po1g strain	Yeastern Biotech Co., Ltd., Taiwan
Oligonucleotides			
AMY_SfiI_F	AAGGCCGTTCTGGCC ATGAAGGTGCTCGCC		This study
AMY_KpnI_R	AAGGTACC CTAGTGGTGGTGGTGGTGGTGC		
r-t_actin_F	CGAGCGAATGCACAAGGA	Real-time analysis—copy number determination	Celińska and Grajek (2013)
r-t_actin_R	GAGCGGTGATCTTGACCTTGA		
r-t_AMY_F	GTAACAACGTGGGAATCCGAAT		This study
r-t_AMY_R	CCCTGGCCGTTCGAAGTAG		

### DNA manipulation and vector construction

Standard molecular biology techniques were used throughout this study (Sambrook and Russell 2001). The sequence of the *Amy1* gene from *S. oryzae* (gb|HQ158012.1) was codon-optimized for expression in *Y. lipolytica* (gb|KP027641) at GenScript Facility (GenScript Inc., Piscataway, USA). Vectors and oligonucleotides used in this study are summarized in Table 1. Restriction enzymes, shrimp-alkaline phosphatase, and DNA molecular markers for electrophoresis were all purchased from Thermo Fisher Scientific Inc. (Waltham, MA, USA). DNA T4 ligase was obtained from New England Biolabs (UK). DNA Taq polymerase was purchased from Qiagen (Germany). PCR reactions were set up in a Veriti® Thermal Cycler (Applied Biosystems), using *AMY_SfiI_F* and *AMY_KpnI_R* primers (0.5 μM each) and approx. 50 ng of DNA template, in a final volume of 25 μL, using the following temperature profiles: 94 °C for 5 min; (94 °C for 30 s, 56 °C for 30 s, 72 °C for 90 s) 25×; 72 °C for 3 min. All the reactions were set up according to the protocols recommended by the suppliers. The following steps were carried out with the use of an appropriate kit from A&A Biotechnology (Gdynia, Poland): genomic DNA isolation from yeast (Genomic Mini AX yeast kit), plasmid DNA isolation (Plasmid Mini kit), and DNA fragment purification from agarose gel (Gel Out kit). Prior to transformation, the *Amy1* gene sequence was verified through sequencing (Genomed sequencing facility, Poland).

### Transformation and selection of positive clones

*E. coli* JM109 competent cell preparation and heat-shock transformation were carried out according to standard protocols (Sambrook and Russell 2001). Positive clones were selected on LB + ampicillin agar plates and screened through plasmid mini-preparation and endonuclease digestion. Preparation of *Y. lipolytica* Po1g competent cells and transformation with the expression cassette were completed according to the protocol supplied by the manufacturer of the YLEX Expression kit (Yeastern Biotech Co., Ltd., Taiwan). Positive clones were selected on YNB plates. Phenotype verification was carried out on YPS medium (g/L): yeast extract, 10; bactopeptone, 20; glucose, 20; agar, 20; and soluble starch, 10; after 24-h growth, the biomass was scraped and the 5 % iodine solution (I_2_ in KI) was poured onto the plate to visualize translucent zones.

### Estimation of the recombinant gene copy number

The number of the recombinant *Amy1* gene copies integrated with the host genome was estimated through real-time quantitative PCR analysis. The reaction was carried out using Real-Time 2xPCR Master Mix SYBR kit A (A&A Biotechnology, Gdynia, Poland). The reactions were set up according to the manufacturer’s protocol in Applied Biosystems 7500 device. Real-time PCR primers targeting the *Amy1* gene (*r-t_AMY_F* and *r-t_AMY_R*) and the actin-coding gene (*r-t_actin_F* and *r-t-actin-R*), used as an endogenous control, were designed with Primer Expert Software (Applied Biosystems). The following temperature profiles were applied: 94 °C 5 min; (94 °C 30 s, annealing temperature according to Primer Expert 15 s, 72 °C 45 s) 40×; 72 °C 5 min; melt curve: 94 °C 15 s, 60 °C 60 s, 95 °C 30 s, and 60 °C 15 s. Fluorescence from SYBR® Green was measured at the end of the elongation step. Samples were analyzed in triplicate. Data analysis was carried out according to the standard ΔΔC_T_ method (Livak and Schmittgen 2001).

### Amylase activity assay

The amylase activity was determined by measuring the amount of reducing sugars released after incubation with starch. One milliliter of liquefied starch solution (1 %; *w*/*v*) in acetate buffer (100 mM, pH 5.0) was used as the substrate. The reaction was initiated at the enzyme preparation addition (0.4 mL) and was continued for 30 min at 40 °C. The concentration of reducing sugars was determined according to the Nelson-Somogyi method (Nelson 1944) versus a standard curve prepared with glucose. The concentration of background sugars contained within the enzymatic preparation was assessed and taken into account in the calculations. All the measurements were done in three technical replicates. One activity unit was defined as the amount of an enzyme that released reducing sugar ends equivalent to 1 μmol of glucose per 1 min under the specified assay conditions.

For assessing the optimal assay temperature and pH, the assay conditions were modified by incubating the reaction mixture at 25, 30, and 40 °C and dissolving starch in either 100 mM acetate buffer (for pH 4.0, 4.5, 5.0, and 5.5) or 100 mM phosphate buffer (for pH 6.0 and 6.5). Ca^2+^ dependence of the α-amylase was verified by comparison of the enzyme activity toward the substrate with and without provision of CaCl_2_ at 2 mM in the acetate buffer, pH 5.0.

Amylase activity was determined in the culture medium and inside the yeast cells immediately after the collection of the samples. For the extracellular amylase activity, after separation of the biomass through centrifugation (4234×*g*, 4 °C, 10 min), raw medium was added into the reaction mixture, without any preprocessing. The protein concentration was determined by the method described by Bradford (1976), using bovine serum albumin (BSA) as a standard.

### Preparation of protein extracts

Protein extracts were prepared by resuspending the cellular pellets in breaking buffer (0.1 M sodium phosphate buffer, 5 μM DTT, 1 mM PMSF, 5 % glycerol) with glass beads (Sigma-Aldrich Co., USA) and disruption of the cells by repeated cycles (5×) of mixing at 30 strokes/s for 1 min in a Mixer Mill MM400 (Retsch) and incubation on ice for 1 min. The cellular debris were then separated by centrifugation (24,652×*g*, 4 °C, 10 min).

### Testing various starch types for digestibility by the new amylase

Digestibility of 11 starch species, of various botanical origin, was tested. The following starch types were analyzed: soluble potato, potato Z1, potato Z2S, waxy potato, waxy rice, waxy corn, corn, tapioca, pea, amaranth, and wheat. The starches were suspended in acetate buffer (100 mM, pH 5.0) and either liquefied through autoclaving or provided into the reaction mixture as a granular substrate. For the liquefied substrates, the reaction lasted 30 min, and 0.368 mAU was added per reaction, while for the native, granular substrates, the reaction was continued up to 1 h, and 38.6 mAU was added into the reaction mixture. Digestion was followed by the Nelson-Somogyi protocol for determination of reducing sugars.

### Bioreactor cultivations

Bioreactor cultivations were carried out in BIOSTAT® A plus (Sartorius) stirred tank bioreactors, with a total volume of 5 L and culture medium volume of 2 L. The culture medium (YPG (g/L): yeast extract, 10; bactopeptone, 20; glycerol, 100) was inoculated at 10 % (*v*/*v*) with 22-h-old YPG culture. pH was adjusted to 5.5 by automatic addition of alkali (20 % NaOH); no acid was fed into the bioreactor. The temperature was maintained at 30 °C throughout the culture. Stirring and aeration were automatically adjusted to maintain oxygen saturation of the culture at 30 %. Foam formation was minimized by automatic addition of Antifoam 204 (Sigma-Aldrich). Culture parameters were automatically monitored with BioPAT® MFCS SCADA. Dry cellular weight (DCW) was determined through a gravimetric method.

### Enzyme purification through affinity chromatography

Extracellular proteins were precipitated with ammonium sulfate (to the final saturation of 80 %, at 4 °C) for several hours. After centrifugation (4234×*g*, 4 °C, 45 min), the proteins were resuspended in binding buffer (phosphate buffer, 20 mM, pH 7.4; NaCl, 0.5 M; imidazole, 20 mM), filtered through a 0.45-μm syringe filter (Millex, Millipore), and loaded onto the ÄKTA FPLC system (ÄKTA Pharmacia GE FPLC) equipped with a HisTrap HP column (5 mL, GE Healthcare), with Ni^2+^ ions immobilized on sepharose. The purification procedure was carried out under increasing gradient of elution buffer (phosphate buffer, 20 mM, pH 7.4; NaCl, 0.5 M; imidazole, 0.5 M) at the flow rate of 5 mL/min. Fractions were immediately analyzed for the amylase activity and subjected to sodium dodecyl sulfate-polyacrylamide gel electrophoresis (SDS-PAGE), to identify the enzyme-enriched fraction and assess its purity.

### Analytical procedures

#### Electrophoresis techniques

DNA fragments were resolved in 1 % agarose gels in TBE buffer and stained with ethidium bromide. SDS-PAGE was carried out according to the standard method of Laemmli (1970); the proteins were resolved in 15 % denaturating polyacrylamide gel. For zymography, the protein extracts from the pellets, the ammonium sulfate (80 %) concentrated proteins from the medium, or ÄKTA-purified α-amylase was resolved in native 10 % PAA gel, supplemented with 1 % of liquefied soluble starch, and added prior to polymerization. After electrophoresis, the gels were incubated overnight in acetate buffer (100 mM, pH 5.0) with 2 mM CaCl_2_ and 10 mM NaCl. The gels were revealed by incubation with 5 % iodine solution.

#### Scanning electron microscopy

Prior to SEM analysis, the samples of digested and nondigested starch granules were subjected to repeated washing with 20 mM NaOH and ddH_2_O, as previously described (Białas et al. 2013), and subsequently lyophilized. Lyophilization was carried out in vials in Christ Beta lyophilizator (Christ, Germany), applying the following parameters: initial freezing −35 °C; drying: condenser −35 °C, shelf 15 °C, pressure 0.22 mbar, time 40 h; and final drying: shelf 20 °C, pressure 0.18 mbar, time 8 h. Samples were stored in a desiccator until analysis. The samples were placed on specimen stubs covered with double-sided adhesive tape and sprayed with gold particles (99.9 %) in sputter coater Bal-Tec SCD 050 (Balzers, Liechtenstein). The granules were observed and photographed using a scanning electron microscope (Zeiss EVO 40, Carl Zeiss AG, Oberkochen, Germany).

## Results

### Cloning and sequence analysis

The cDNA sequence encoding the α-amylase (*Amy1*) gene from *S. oryzae* (gb|HQ158012.1) consists of 1458 bp encoding 486 amino acid residues. This sequence was codon-optimized for cloning and expression in *Y. lipolytica* (gb|KP027641), to avoid codon usage incompatibility, which we have encountered in our previous study (Celińska and Grajek 2013). Codon-optimized sequence was 100 % identical in primary amino acid structure with the original sequence from *S. oryzae*. Additionally, the *Amy1* sequence was tagged with a 6xHis-tag, at the C-terminus, to enable easier purification in an IMAC system. The calculated molecular weight of the protein was 53.9 kDa (which was also confirmed by SDS-PAGE in this study), and the theoretical isoelectric point, p*I*, was 4.92 (ExPASy, ProtParam, http://web.expasy.org/protparam/). The sequence was also found to possess a 17-amino-acid signal peptide at the N-terminus (PrediSi, http://www.predisi.de/predisi/). The signal peptide appeared to be operable in *Y. lipolytica*, when the *Amy1* gene was cloned in pYLEX vector, not equipped with *Y. lipolytica* secretion signal (data not shown). Functionality of signal peptides from phylogenetically distant organisms in *Y. lipolytica* has been also reported for other proteins (Park et al. 1997; Yuzbashev et al. 2012). The amino acid sequence of the AMY protein was 70 % similar to α-amylase from *Anthonomus grandis* (gb|AAN77138.1|) and an unknown protein from *Dendroctonus ponderosae* (gb|AEE61654.1) and 68 % similar to amylase A and amylase B protein sequence from *Ips typographus* (gb|ADQ54210.1 and gb|ADQ54211.1). The structure of the AMY protein, regarding the organization of the domains, was typical for the other amylases, with an “AmyAc_bac_euk_AmyA” domain (alpha-amylase catalytic domain found in bacterial and eukaryotic alpha-amylases) and a “C-terminal-all-beta” domain. Importantly, the C-terminal binding domain is considered to be responsible for adsorption of the enzyme on the surface of starch granules (Jespersen et al. 1991). The occurrence of this domain is associated with the ability to digest raw starch granules, as it was reported for fungal glucoamylases from *Rhizopus* and *Aspergillus* or bacterial β-amylases (Sarikaya et al. 2000). Plant β-amylases, lacking the characteristic “C-terminal-all-beta” sheet domains, also lack the ability to digest raw starch.

In this study, the *Amy1* gene was also cloned in pYLSC vector, which includes additional signal peptide encoded within the vector’s sequence and drives the recombinant gene expression from a hp4d strong hybrid promoter. The vector targets insertion of the cloned gene in the pBR322 docking platform that is contained within the compatible *Y. lipolytica* Po1g strain’s genome in a single copy. We screened the obtained transformant strains for the copy number of the recombinant *Amy1* gene, using real-time PCR technique. The selected strain, *Y. lipolytica* 1.18, with an estimated four copies of the recombinant *Amy1* gene (the relative quantitation value ΔΔC_T_ was 4.17 calculated vs. Po1g parental strain), was further used in this study.

### Production of the α-amylase in *Y. lipolytica* 1.18 strain and purification through affinity chromatography

**Fig. 1:**
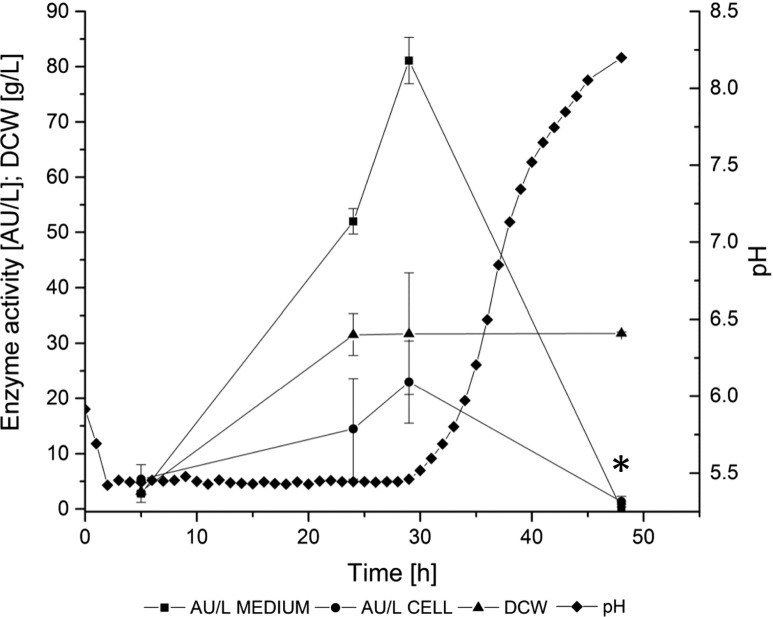
α-Amylase production in batch bioreactor cultures of *Y. lipolytica* 1.18 strain. The cultures were carried out in a 5-L bioreactor, with a working volume of 2 L, in YPG-rich medium, at pO_2_ 30 %; pH was adjusted to 5.5 by the addition of alkali. Amylase activity was determined in raw medium (*closed squares*) and protein extract from the cells (*closed circles*), according to the Nelson-Somogyi method. [DCW]: dry cellular weight was determined by gravimetric method (*closed triangle*). *x-axis*: time of culture [h]; *y-axis left*: [AU/L]: activity units per liter of the culture medium; [DCW] expressed in [g/L]. *y-axis right*: pH (*closed diamonds*). *Asterisk* marks a decrease in the [AU/L] parameter attributed to elevated pH. The culture was carried out in three independent runs. All the measurements were done in three technical replicates for each culture. *Error bars* indicate ± SD from the three independent runs

The recombinant α-amylase was produced by the *Y. lipolytica* 1.18 strain in a growth-dependent manner, without the need for induction with starch. The culture medium contained glycerol as the sole carbon source. Elimination of glucose, or any other sugar, from the medium recipe, was convenient for the amylase assay, as the background sugar level was relatively low. Importantly, most of the α-amylase activity was detected in the culture medium, confirming that the protein was efficiently targeted to the secretory pathway of the yeast cells (Fig. 1), which greatly facilitated the subsequent purification step. The recombinant α-amylase activity in the culture medium was observed after only 29 h of culturing, immediately after entering the stationary phase of growth. The production of the recombinant α-amylase secreted into the culture medium reached the maximum value of 81 AU/L, at the volumetric and specific productivity of 2.8 AU/L h and 2.56 AU/gDCW, respectively (Table 2). A minor fraction of the α-amylase activity was detected inside the cells (23 AU/L, 0.79 AU/L h, and 0.72 AU/gDCW).

**Table 2:** Productivity and specific productivity of the recombinant α-amylase production by *Y. lipolytica* 1.18 strain in bioreactor batch cultures

	[AU/L h] ± SD	[AU/gDCW] ± SD	[AU/gDCW h] ± SD
Medium			
5 h	0.56 ± 0.07	0.88 ± 0.11	0.18 ± 0.02
24 h	2.17 ± 0.09	1.65 ± 0.07	0.07 ± 0.003
29 h	2.8 ± 0.14	2.56 ± 0.13	0.09 ± 0.004
48 h	0 ± 0.01	0.00 ± 0.02	0.00 ± 0.0005
Pellet			
5 h	1.1 ± 0.51	1.70 ± 0.81	0.34 ± 0.16
24 h	0.60 ± 0.37	0.46 ± 0.28	0.02 ± 0.01
29 h	0.79 ± 0.25	0.72 ± 0.23	0.025 ± 0.008
48 h	0.03 ± 0.02	0.031 ± 0.04	0.00 ± 0.0009

**Fig. 2:**
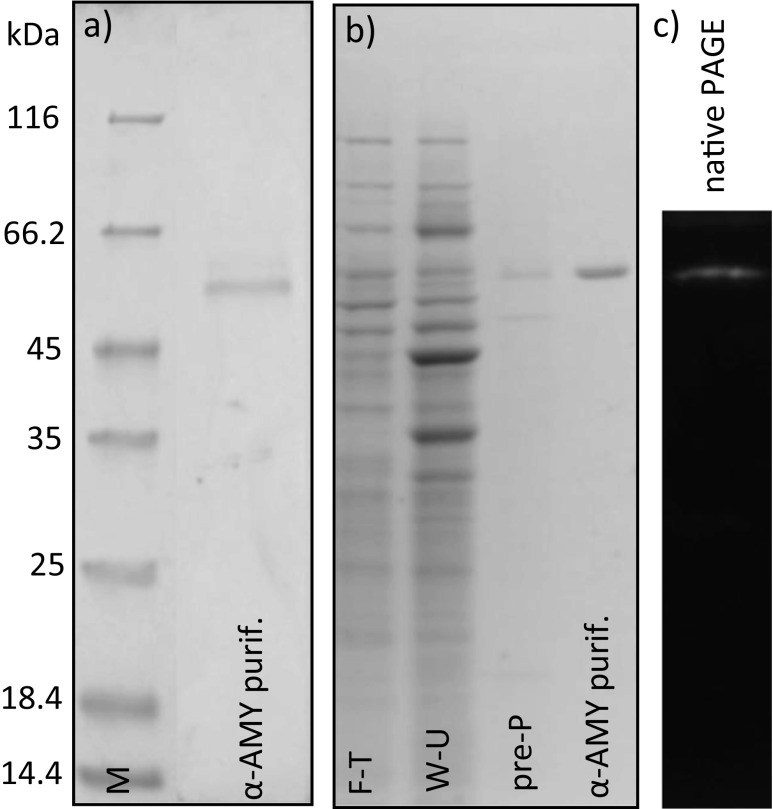
SDS-PAGE and native PAGE separation of protein samples. **a** SDS-PAGE separation of purified α-amylase after ammonium sulfate precipitation and affinity chromatography with a molecular mass marker (*M*; unstained protein molecular weight marker, Thermo Scientific). **b** SDS-PAGE electrophoretic separation of fractions after affinity chromatography; *F-T* flow-through, *W-U* wash-unbound, *pre-P* pre-peak with low α-amylase activity, *α-AMY purif.* fraction with the highest α-amylase activity. **c** Native PAGE electrophoretic separation of α-amylase-containing enzymatic preparation; the gel contained 1 % of liquefied starch; after separation, the gel was incubated O/N in Ca^2+^/NaCl acetate buffer and afterward stained with iodine

Purification of the recombinant α-amylase was carried out through precipitation of total protein contained within the culture medium with ammonium sulfate, followed by affinity chromatography. Overall 25-fold purification was achieved with 5 % yield, resulting in 2.54 mg of purified α-amylase activity, in the two-step procedure (Table 3). It was possible to purify the enzyme to apparent homogeneity, visualized by SDS-PAGE, through this simple procedure. The purification process was monitored through SDS-PAGE (Fig. 2). The purified recombinant α-amylase migrated as a single band of approximately 53 kDa, as predicted from the translated nucleotide sequence. Native PAGE analysis of both the medium protein precipitate and the purified α-amylase fraction revealed the presence of a single amylolytic isozyme, with no interfering activities present.

**Table 3:** Purification of the recombinant α-amylase from *Y. lipolytica* 1.18 bioreactor culture

Purification step	Activity [AU/L]	Volume [mL]	Total activity [AU]	Protein [mg/mL]	Total protein [mg]	Specific activity [AU/mg]	Yield [%]	Purification factor [fold]
Crude medium	84.24	700	58.97	1.84	1290.06	0.05	100	1.00
Ammonium sulfate 80 % + affinity chromatography	481.67	6	2.89	0.42	2.54	1.14	5	24.91

### Characterization of the purified amylase

**Fig. 3:**
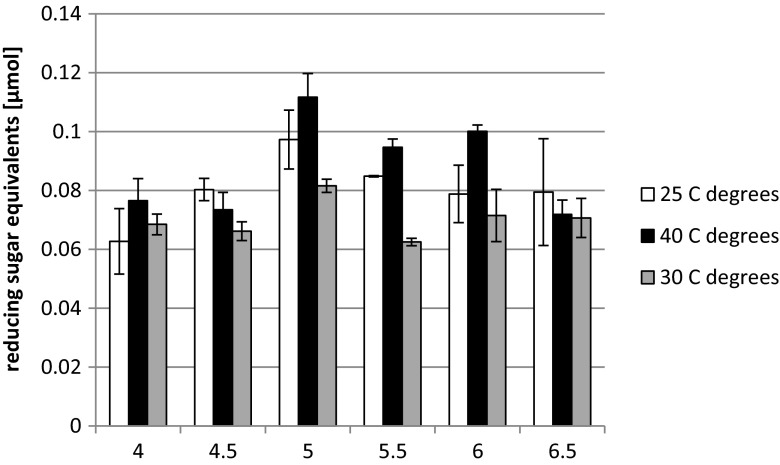
α-Amylase activity at different pH values and temperatures. Liquefied starch solution in 100 mM acetate buffer (for pH 4.0, 4.5, 5.0, 5.5) or 100 mM phosphate buffer (for pH 6.0 and 6.5) was used as the reaction substrate. The assay was carried out according to the Nelson-Somogyi method, under varied thermal conditions: 25, 30, and 40 °C (°C marked as “C degrees” in the figure). *y-axis*: reducing sugar equivalents released under the assay conditions in micromoles. *Error bars* indicate ± SD from triplicate measurements

The purified recombinant α-amylase was tested for optimal pH and temperature and also for the Ca^2+^ ion requirement. The optimal conditions for the α-amylase activity were pH 5.0 and temperature of 40 °C (Fig. 3). The α-amylase studied here did not show any obligate requirement for metal ions, as no apparent increase in activity was observed with Ca^2+^ cation provision (0.82 ± 0.06 vs. 0.79 ± 0.05 of released reducing sugar equivalents with and without Ca^2+^, respectively; ±SD).

**Fig. 4:**
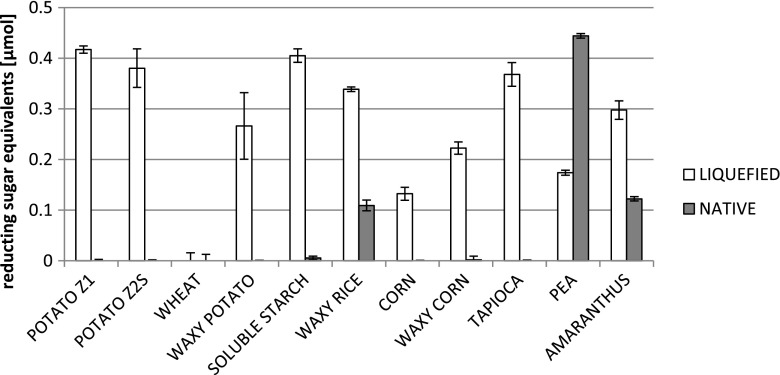
Digestibility of different starch species by recombinant α-amylase enzymatic preparation. Starch was provided as 1 % solution in 100 mM acetate buffer, pH 5.0. The reaction mixture was incubated at 40 °C for 30 min with 0.386 mAU of α-amylase or 1 h with 36.8 mAU of α-amylase for liquefied and native substrates, respectively. *y-axis*: reducing sugar equivalents released under the assay conditions in micromoles. *Error bars* indicate ± SD from triplicate measurements

Eleven species of raw starch granules of different botanical origin were tested for digestibility by the purified α-amylase (Fig. 4). As expected, the substrates varied significantly in the degree of decomposition by the enzyme, which obviously resulted from varying sizes and structures of the granules, but also from different contents of amylose/amylopectin in the respective substrates. Surprisingly, the highest decomposition degree was observed for the pea starch, followed by amaranth, waxy rice, and waxy corn (0.44 ± 0.004, 0.12 ± 0.004, 0.11 ± 0.01, and 0.01 ± 0.0001 μmol of reducing sugar equivalents, respectively; ±SD), with no detectable degradation of granular starch originating from potato, tapioca (both starches characterized by a high degree of amylose polymerization), wheat, or amylose-containing corn. It has been reported that pea flour is a strong repellent of *S. oryzae*, which makes it a promising protecting agent of stored rice and wheat grains (Pretheep-Kumar et al. 2004). However, it has been observed that pure pea starch is not the repelling agent, but rather pea proteins, contained in the pea flour, exert the repelling effect. Our studies indicate that pea starch in its native, granular form can be efficiently decomposed by the digestive enzyme synthesized in the alimentary tract of *S. oryzae*.

With respect to decomposition of liquefied starch, most of the tested substrates were digested on a similar level (0.26 to 0.41 μmol of reducing sugar equivalents), apart from waxy corn, corn, and pea, which were digested to a slightly smaller degree (0.13 to 0.22 μmol of reducing sugar equivalents) (Fig. 4). Surprisingly, wheat starch was not digested even in liquefied form, which obviously indicates the presence of some proteinaceous inhibitors.

**Fig. 5:**
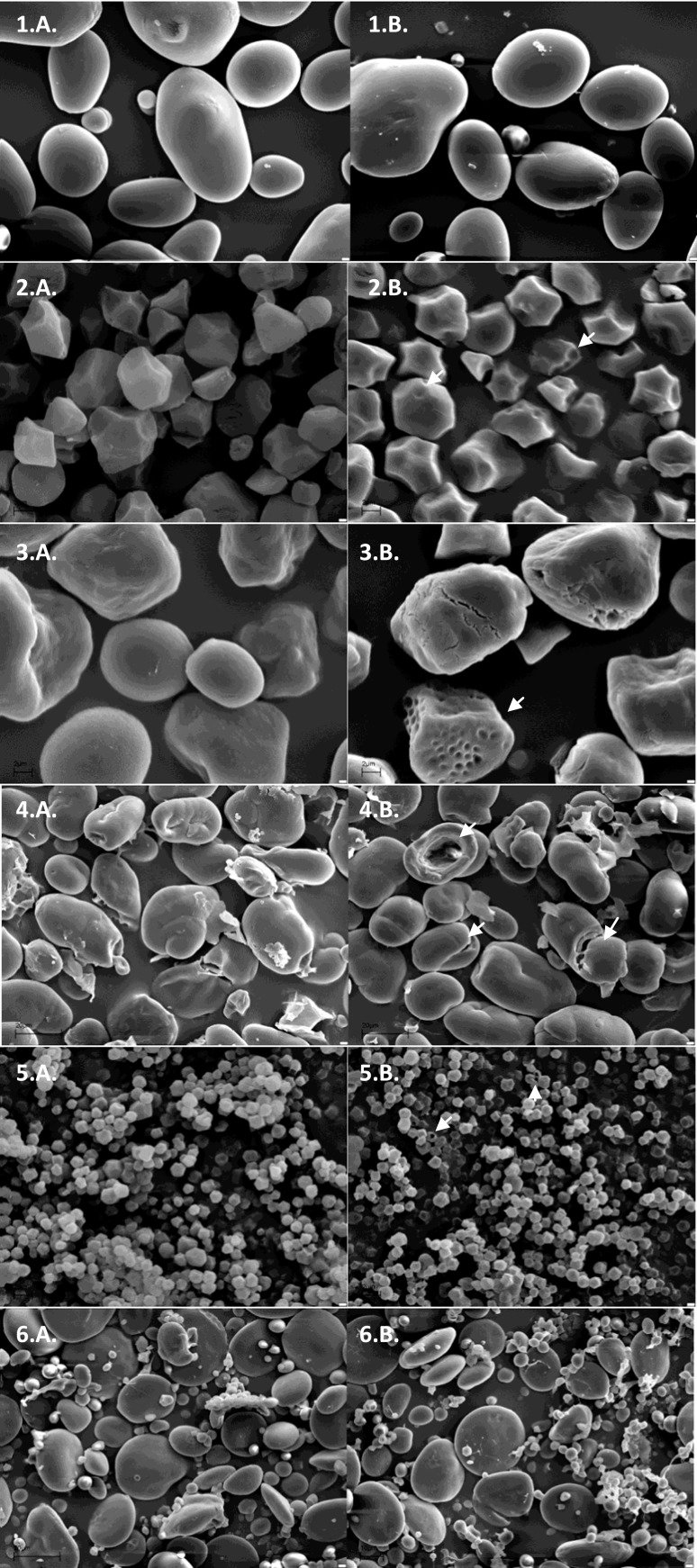
Scanning electron micrographs of various starch granules digested with the recombinant α-amylase. Digestion was carried out according to the procedure described in the section “Testing various starch types for digestibility by the new amylase *,*” and samples were prepared according to the procedure described in the section “Scanning electron microscopy *.*” Starch types: *1*. potato, *2*. waxy rice, *3*. waxy corn, *4*. pea, *5*. amaranth, *6*. wheat. *A.* nontreated sample, *B,* treated sample. *White arrows* indicate signs of α-amylase action

SEM micrographs confirmed the observations from the enzymatic assay (Fig. 5). Pea and amaranth starch granules appeared like drilled through, presenting extensive erosion in their interior, with no formation of pits and craters on the surface, showing centripetal type of degradation by the α-amylase. Waxy corn and waxy rice granules were digested by the amylase by creating small shallow pits and crater-like areas on the surface of the granules. Wheat and soluble potato starch remained intact after the α-amylase treatment.

## Discussion

Amylolytic enzymes are one of the most important industrially relevant enzymes, accounting for approximately 30 % of the world’s production of enzymes (van der Maarel et al. 2002). Despite of the wide distribution of amylases among plant, animal, and microbial species, only fungal and bacilli enzymes constitute a significant contribution to the world’s market. Other sources are significantly less explored. For most insect species studied to date, information on the raw-starch-digesting enzymes has been very scarce. The main factor limiting the progress within this field is a laborious, inefficient, and multistep procedure for the isolation and purification of enzymes from small animals. Heterologous overexpression offers the possibility to surpass these constraints.

In this study, *Y. lipolytica* was exploited as the host for expression of insect (*S. oryzae*) α-amylase, to investigate its potential usefulness in raw starch digestion. *Y. lipolytica* Po1g strain was transformed with pYLSC-*Amy1* expression cassette, intended for integration into the host genome. Integration of the expression cassette with the host genome offers a significant advantage over the episomal expression platforms. This is particularly true for *Y. lipolytica*, as really potent episomal plasmids are currently just being developed (Liu et al. 2014). Integrative expression cassettes are more stable and eliminate the necessity of selection pressure maintenance, which is of significant importance during large-scale and/or prolonged cultivations in complex, nondefined media. Our complementary experiments showed significant improvement in the AMY protein production by the 1.18 strain in the rich medium (YPG), when compared to the production in the minimal medium, enabling selection pressure maintenance (YNB, with glycerol at 20 g/L as a carbon source; data not shown). Therefore, the bioreactor cultivations were all carried out in the rich YPG medium.

To select a single clone for the production of the recombinant α-amylase, the transformants were screened for estimated copy number of the heterologous *Amy1* gene, using real-time PCR technique. As demonstrated by Nicaud et al. (2002), the heterologous gene copy number was a strong determinant of the amount of an enzyme produced, contributing to up to 8-fold increase in the enzyme activity, solely due to gene amplification. The technique applied in this study indicated the presence of 4 copies of the *Amy1* gene per genome of *Y. lipolytica* 1.18 strain. However, further studies are necessary to confirm this observation and reveal the mode of the cassette integration (tandem or random, out of site), as integration of higher number of copies is rather surprising in the strain Po1g-pYLSC1 system. Although recent papers state that *Y. lipolytica* is known to preferentially use the nonhomologous end-joining mechanism for DNA repair over homologous recombination (Kretzschmar et al. 2013; Verbeke et al. 2013), which could potentially explain the integration of a higher number of copies of the expression cassette than the number of potential integration sites (single pBR322 docking platform per Po1g strain’s genome), it was also reported that out-of-site integrations are rare events in *Y. lipolytica* (Madzak et al. 2000). In the first report describing hp4d promoter, similar pBR-based vectors were used, several tens of transformants were analyzed by Southern blotting, and only one example of a double tandem integration (at the intended pBR docking platform) was identified (Madzak et al. 2000). According to the review by Barth and Gaillardin (1996), when using a sufficiently large region of homology (more than 300 bp on each side—which is significantly less than in the pYLSC1 vector), a single complete copy of the expression cassette integrates at the chosen site in more than 80 % of the cases, while the remaining events include multiple tandem integrations, gene conversions, and out-of site integrations.

The recombinant α-amylase production by the *Y. lipolytica* 1.18 strain during the bioreactor batch cultivations was very rapid. As indicated in Fig. 1, the peak value in the recombinant α-amylase activity was reached immediately after entering the stationary phase of growth. Importantly, during the next time-point interval, we observed a significant decrease in the enzyme activity. The observed drop in the recombinant α-amylase activity could be possibly attributed to a rapid increase in the pH value. Probably, the high pH value was detrimental to the heterologous α-amylase activity assessment, since at pH ~ 8.0, the activity was not detectable, both in the cell and in the medium. Noteworthy, we were still able to recover the α-amylase from the end-point culture medium after the purification procedure, through ammonium sulfate precipitation and affinity chromatography, which demonstrates that the protein was still present in the medium. Also, previous reports on heterologous enzyme production within the *Y. lipolytica*-hp4d promoter-based expression systems showed continued accumulation of the proteins during the stationary growth phase rather than a peak value followed by a decrease in activity (Madzak et al. 2005; Yang et al. 2010a). According to the characteristics provided by Madzak et al. (2004), hp4d recombinant promoter is almost independent from environmental conditions and is able to drive a strong expression in virtually any medium, promoting growth-phase-dependent gene expression. It was found that hp4d-driven heterologous gene expression occurs mostly at the beginning of the stationary phase (Nicaud et al. 2002), which we have also observed in our study. However, the increase in the pH value obviously hampered further accumulation of the recombinant α-amylase.

When compared with the literature data, the α-amylase activity was detected relatively fast. Huang et al. (2011), using the same expression system, observed peak activity of esterase after 72 h of fed-batch culture. A similar time (60–72 h) was required for the maximal invertase production by recombinant *Y. lipolytica* strains (Förster et al. 2007; Lazar et al. 2011). Boisramé and Gaillardin (2009) observed production of a recombinant β-1,6-glucanase in *Y. lipolytica* Po1g strain after 60 h of culturing. In our complementary experiments, we used minimal, defined medium (YNBG; glycerol at 100 g/L instead of glucose) in the bioreactor cultures, which resulted in the peak value in the heterologous α-amylase activity after 72 h of culturing (data not shown). Therefore, the rapid character of the culture could be attributed to the use of a rich, nonselective medium. Correspondingly, a rapid production trend has been observed for recombinant *Y. lipolytica* strain expressing heterologous fatty acid hydroperoxide lyase, where the maximum of the enzyme activity was observed after 24 h of culturing (Santiago-Gómez et al. 2007). While in the earlier study of Bourel et al. (2004), the peak activity of fatty acid hydroperoxide lyase expressed in *Y. lipolytica* was observed in the stationary phase of growth (between 86 and 92 h of culturing).

Most of the produced α-amylase was secreted into the culture medium; however, the obtained amount of the enzyme remains to be further optimized. The amount of fatty acid hydroperoxide lyase expressed in *Y. lipolytica* strain in rich medium was 2500 AU/L, but the enzyme was retained within the cells (Santiago-Gómez et al. 2007); 4000 AU of rice α-amylase per liter of crude medium was reported by Park et al. (1997). This result was further improved by optimization of the culturing mode (Chang et al. 1998; Kim et al. 2000; discussed below). In another paper, heterologous expression of thermostable microbial α-amylase in *Y. lipolytica* host has been reported (Yang et al. 2010a). The enzyme was produced at a high yield of 730 AU/L. Comparison with the authors’ previous studies, where the same gene was expressed in *P. pastoris* (Yang et al. 2010b) and *E. coli* (Yang and Liu 2007), proved *Y. lipolytica*-pYLSC1 host to be the superior expression system over the other two analyzed. Förster et al. (2007) and Lazar et al. (2011) produced invertase in recombinant *Y. lipolytica* cells, but the reported activity units per milliliter values differed significantly, with 105 and 589 U/gDCW of extracellular fraction, respectively. In the study by Lazar et al. (2011), very high and rapid invertase production was observed (300 to 442 U/L h), but the intracellular enzyme fraction was significantly higher (up to 3736 U/gDCW). Although the ultimate values of the results cited above cannot be directly compared, due to different enzymatic activities being determined, it appears that the potential of *Y. lipolytica* was not fully explored in this study, and further research on improvement of the recombinant α-amylase production will be carried out. As demonstrated by Nicaud et al. (2002), fed-batch cultures were highly beneficial for overexpression of heterologous proteins in *Y. lipolytica* strains, as illustrated by 7.9-fold or 11-fold increase in lipase and leucine amino peptidase production, respectively, when compared to batch cultures. In another study, rice α-amylase was efficiently produced in *Y. lipolytica* transformant using cyclic fed-batch culture mode, reaching high cell density culture (Chang et al. 1998). Due to intensification of the culturing process, it was possible to obtain as high volumetric productivity of the enzyme as 1960 AU/(L h). In the same paper, the authors describe fed-batch culture, where 31,200 AU/L of α-amylase was obtained. The rice α-amylase production process was further improved in the following study by the same research team (Kim et al. 2000). Using a single feeding step with concentrated substrate into the batch culture, the authors obtained a high cell density culture of the recombinant *Y. lipolytica* (over 100 g DCW/L) with the final α-amylase activity of 88,000 AU/L.

Extracellular localization of the recombinant proteins is highly advantageous for the subsequent purification strategies. In this study, due to majority of the enzyme being secreted into the culture medium, we were able to apply a relatively simple two-step purification procedure. Through ammonium sulfate precipitation followed by affinity chromatography, it was possible to purify the enzyme to apparent homogeneity, with 25-fold purification factor at 5 % yield. Depending on the microbial producer and the applied purification method, RSD amylases could be purified by 2.8- or 8.5-fold at 12 or 60.5 % yield from *Aspergillus* sp. (Okolo et al. 2001), 6-fold at 38 % yield from *Bacillus licheniformis* (Božić et al. 2011), 5.4-fold at 72.9 % yield from *Rhizopus* sp. (Morita et al. 1998), or by 34-fold at 6.6 % yield from *Bacillus* sp. (Liu and Xu 2008). In a recent paper, Kumar and Khare (2012) in a three-step purification procedure obtained 76-fold purification at 52 % yield of halophilic α-amylase from its native producer, *Marinobacter* sp. Single step purification, through in-batch addition of affinity resin, was applied to β-1,6-glucanase secreted into the medium by recombinant *Y. lipolytica*; however, no purification factor and yield were reported (Boisramé and Gaillardin 2009). Santiago-Gómez et al. (2007) purified fatty acid hydroperoxide lyase through extraction of the enzyme from the cells with Triton X-100R (2 %) followed by IMAC chromatography. The initial amount of the enzyme in crude extract was 2500 AU/L, and after purification of 29 AU/L, resulting in 28.1 mg of the purified enzyme. Rice α-amylase was purified from the culture medium of recombinant *Y. lipolytica* by ammonium sulfate precipitation at 75 % saturation and subsequently subjected to β-cyclodextrin affinity chromatography, resulting in 72-fold purification at 47 % yield (Park et al. 1997). In a study by Madzak et al. (2005), heterologous laccase was produced in *Y. lipolytica*, and the purification factor after the five-step purification process (including ultrafiltration of FPLC chromatography) was of 74-fold, with a recovery of 26 %, yielding 0.7 mg of purified recombinant laccase. Heterologous α-amylase from *T. fusca* produced in *Y. lipolytica* was purified 10-fold at 21.87 % yield, using a three-step procedure (Yang et al. 2010a). Lazar et al. (2011) concentrated and purified invertase produced by *Y. lipolytica* by approximately 5- to 10-fold through ultrafiltration. In general, ultrafiltration appears to be one of the most efficient methods of protein concentration from the medium prior to chromatography step, as the reported purification factors and yields compare favorably with the ammonium sulfate-based strategies, e.g., 400-fold purification at 64 % yield from *Bacillus firmus* (Gawande et al. 1999), 141-fold purification at 78 % yield from *Cryptococcus* sp. (Iefuji et al. 1996), or 738-fold purification at 68 % yield from *Klebsiella pneumoniae* (Gawande and Patkar 2001). In this study, a significant loss in the amount of the enzyme purified from the medium was attributed to low efficiency of the ammonium sulfate precipitation step.

The purified α-amylase was subsequently characterized with respect to some of its main characteristics. The optimal conditions for the α-amylase activity were pH 5.0 and temperature of 40 °C. The optimum activity of amylolytic enzymes around pH of 4.5–5.0 is highly desirable, since in the starch industry, the natural pH of starch slurry is usually around 4.5 (Sivaramakrishnan et al. 2006). Most of the commercial bacterial α-amylases are highly active at pH 6.5 and are unstable in an acidic environment, thus causing the necessity of the slurry pH adjustment (Sajedi et al. 2005). Employment of amylolytic enzymes that are stable and highly active under acidic conditions could simplify the starch processing pipeline. The α-amylase studied here did not show any obligate requirement for Ca^2+^ ions. A similar property was reported for amylase from *Bacillus amyloliquefaciens* (Gangadharan et al. 2009). According to Baker (1987), amylases isolated from *S. oryzae* guts, like a number of typical amylases, are activated by Ca^2+^ and Cl^−^ ions (assayed in acetate buffer pH 5.0, 20 mM NaCl, 0.1 mM CaCl_2_). Ca^2+^ ions are known to exert a stabilizing effect on elevated temperature-treated RSDE, by endowing them with a more compact structure. Similarly, greater thermal stability of RSD amylases has been observed under provision of carbohydrate substrate.

In the pioneering studies by Baker and Woo (1992), α-amylase isozymes isolated from the digestive tract of *S. oryzae* were tested on various substrates, including raw starch granules. It was found that corn and wheat starch granules were rather resistant to the action of amylases, while rice starch was relatively prone to digestion (4- and 6-fold more susceptible, when compared to corn and wheat starch, respectively). However, among commercial starch samples, granular potato starch was the most resistant to α-amylases from *S. oryzae*, which stays in full agreement with our observations. Relative to granular potato starch, activity against other tested starches ranged from 2-fold greater for tapioca starch to nearly 16-fold greater for corn starch, which again complies well with our results, since under applied conditions, tapioca native starch was found to be resistant to enzymatic degradation. Potato starch was also found to be the most resistant against digestion, when treated with α-amylase from *B. amyloliquefaciens* (Sarikaya et al. 2000), while cereal starches (rice, corn, and wheat) were easily decomposed by this enzyme. Corn, wheat, and potato starch granules were tested for digestibility by α-amylase from *Bacillus* sp. YX-1, showing a similar degree of decomposition (57.5, 53, and 45 %, respectively) (Liu and Xu 2008). Importantly, we tested the raw starch digestibility in an assay lasting for only 1 h with relatively small amounts of the enzyme preparation (~40 mAU), while a number of assays were continued for 8 to 12 h, with 50 AU per reaction (Liu and Xu 2008), 12 to 24 h (Sarikaya et al. 2000; AU were not reported), and 12 to 48 h, with 0.5 AU per reaction (Meireles et al. 2009). Significant degradation of the starch granules, especially observed for pea and amaranth starches, in a short time and in the presence of small quantities of the enzyme, demonstrates high potential of the α-amylase to digest nonpretreated, raw substrate. Surprisingly, we have observed that wheat starch has not been digested either in native or liquefied form. High antagonistic activity of an α-amylase inhibitor (WRP-25) in wheat starch toward amylase isozymes isolated from the guts of three insect species (i.e., *S. oryzae*) has been reported by Chen et al. (1992). Detailed studies on the interaction of α-amylase from *S. oryzae* and 0.12 inhibitor from wheat have been reported by Baker (1989). Our results concerning wheat starch indigestibility by the α-amylase from *S. oryzae* could be probably attributed to the presence of the inhibitors in the analyzed substrate.

In conclusion, *Y. lipolytica* appeared to be a good host for the expression and efficient secretion of heterologous α-amylase from phylogenetically distant *S. oryzae*. Bioreactor cultures were very rapid (peak activity at 29 h), but further optimization of the process efficiency will be carried out. Purified α-amylase was most active at slightly acidic conditions (pH 5.0), without obvious requirement for Ca^2+^ ions. Importantly, it was found that the enzyme was able to efficiently digest several types of raw, granular starch, in a short time, which is of great potential for industrial applications. Our further efforts will be focused on the optimization of the α-amylase production process.
